# Acquirements for Admission into the Dental Profession

**Published:** 1865-10

**Authors:** A. Berry


### ACQUIREMENTS FOR ADMISSION INTO THE DENTAL PROFESSION.

BY A. BERRY, D. D. S.

[Read before the Cincinnati Dental Association, Aug. 24th, 1865.]

That a knowledge of anatomy, physiology, pathology, dental therapeutics, chemistry, the theory and practice of operative and mechanical dentistry, is a necessary qualification for the practice of dentistry is too apparent to be denied.

So much does the health and comfort of the patient depend on dental operations being properly performed, that they should never be attempted by the ignorant.

The necessity of thorough preparatory study for the practice of medicine has always been recognized by the medical profession, and by all enlightened communities. From the days of Hippocrates to the present, the doors of the profession have been jealously watched to exclude the uninstructed. In the older states of our country, legal safe-guards were early thrown around the practice of the healing art. In New York, half a century ago, the aspirant to it must study with a reputable physician four years, and be examined and licensed by a legally appointed board of medical men, before he could assume the responsibilities of the profession. At that early period, owing to the financial embarrassments of the country, the expense of attending the few medical schools was quite onerous.

It has been the opprobrium of the dental profession from its earliest history, that in its ranks were many charlatans, there being no criterion by which the public could distinguish them from well educated and honorable members. The veriest ignoramus, with *quantum sufficit* of assurance, had only to say "I am a dentist," and he was a dentist.

Until of comparatively recent date, there being no schools for instruction in dental science, the qualifications for practice were only to be acquired under great difficulties with private preceptors, on whom an immense task was imposed to prepare their students, even in a superficial manner, for the duties of their profession. Although private dental tuition is not to be dispensed with, it can not afford all the necessary advantages.

But, fortunately for our country, the means of obtaining a thorough dental education have been ample for the last quarter of a century. Our dental colleges afford, in this respect, all that could be desired, and at small expense. So there is no longer an excuse for any one attempting to enter the profession without being thoroughly instructed in its principles and practice.

In my humble opinion, it is important that measures be adopted to prevent all not duly and truly prepared from assuming the responsibilities of dentists. The community should be protected against dental empirics by legal enactments. Let the dental societies all over our country resolve, that in future no young applicant shall be received by them as a member, or recognized as belonging to the dental profession, unless he is a graduate of a dental college. The possession of a diploma from such an institution by a young man is prima facia evidence that he has enjoyed facilities for acquiring a good dental education, which he has improved in a creditable manner, while the want of it is good ground for the suspicion that he is a quack.

Instead of such a course being ungenerous toward those desirous of entering the profession, it would be an act of the greatest kindness to them, as well as the public, being promotive of the best interests of all parties concerned.

